# Sex-Based Differences in Adélie Penguin (*Pygoscelis adeliae*) Chick Growth Rates and Diet

**DOI:** 10.1371/journal.pone.0149090

**Published:** 2016-03-02

**Authors:** Scott Jennings, Arvind Varsani, Katie M. Dugger, Grant Ballard, David G. Ainley

**Affiliations:** 1 Oregon Cooperative Fish and Wildlife Research Unit, Department of Fisheries and Wildlife, Oregon State University, Corvallis, OR, United States of America; 2 School of Biological Sciences and Biomolecular Interaction Centre, University of Canterbury, Private Bag 4800, Christchurch, 8140, New Zealand; 3 Electron Microscope Unit, Division of Medical Biochemistry, Department of Clinical Laboratory Sciences, University of Cape Town, Observatory, 7700, South Africa; 4 Department of Plant Pathology and Emerging Pathogens Institute, University of Florida, Gainesville, FL 32611, United States of America; 5 U.S. Geological Survey, Oregon Cooperative Fish and Wildlife Research Unit, Department of Fisheries and Wildlife, Oregon State University, Corvallis, OR, United States of America; 6 Point Blue Conservation Science, Petaluma, CA, United States of America; 7 H.T. Harvey and Associates, Los Gatos, CA, United States of America; Universidad de Granada, SPAIN

## Abstract

Sexually size-dimorphic species must show some difference between the sexes in growth rate and/or length of growing period. Such differences in growth parameters can cause the sexes to be impacted by environmental variability in different ways, and understanding these differences allows a better understanding of patterns in productivity between individuals and populations. We investigated differences in growth rate and diet between male and female Adélie Penguin (*Pygoscelis adeliae*) chicks during two breeding seasons at Cape Crozier, Ross Island, Antarctica. Adélie Penguins are a slightly dimorphic species, with adult males averaging larger than adult females in mass (~11%) as well as bill (~8%) and flipper length (~3%). We measured mass and length of flipper, bill, tibiotarsus, and foot at 5-day intervals for 45 male and 40 female individually-marked chicks. Chick sex was molecularly determined from feathers. We used linear mixed effects models to estimate daily growth rate as a function of chick sex, while controlling for hatching order, brood size, year, and potential variation in breeding quality between pairs of parents. Accounting for season and hatching order, male chicks gained mass an average of 15.6 g d^-1^ faster than females. Similarly, growth in bill length was faster for males, and the calculated bill size difference at fledging was similar to that observed in adults. There was no evidence for sex-based differences in growth of other morphological features. Adélie diet at Ross Island is composed almost entirely of two species—one krill (*Euphausia crystallorophias*) and one fish (*Pleuragramma antarctica*), with fish having a higher caloric value. Using isotopic analyses of feather samples, we also determined that male chicks were fed a higher proportion of fish than female chicks. The related differences in provisioning and growth rates of male and female offspring provides a greater understanding of the ways in which ecological factors may impact the two sexes differently.

## Introduction

Sexual size dimorphism (SSD) is common in a wide range of animal taxa and is thought to result from different selective forces operating on males and females for their different reproductive or ecological roles [1,2]. In many vertebrate species, the evolution of SSD has been linked to social mating systems and sexual selection, with greater SSD observed in polygynous than monogamous species [3,4]. However, these selective forces may also derive from natural selection, and efforts are ongoing to determine the relative importance of these factors [5].

Seabird species show both male- and female-biased SSD, and the direction of dimorphism has been linked to a range of behaviors and other ecological factors. Differences in the magnitude of SSD between certain seabird species may be related to aerial- vs. ground-based displays by males of each species, with female-biased SSD in species where males do aerial displays [6]. Additionally, SSD in mass and wing morphology may result from foraging niche divergence of males and females, perhaps reducing intersexual competition for resources in these central-place foraging species [7].

Two non-exclusive processes may result in SSD: the larger sex may grow faster, or may grow for a longer period of time, or there could be some combination of the two. A range of combinations of growing faster and growing longer have been observed in birds. In several avian species, chicks of the larger sex grow faster and fledge larger (e.g., Yellow-headed Blackbird [*Xanthocephalus xanthocephalus*], [8]; Wandering Albatross [*Diomedea exulans*], [9]). However, in at least one other bird species the sexes grow at similar rates but the larger sex grows for a longer time period (Common Tern [*Sterna hirundo*], [10]

Growth rates are an important component of life history [11], providing mechanisms for a range of ecological trade-offs between fast and slow growth. Faster-growing individuals may out-compete siblings for food and may be less susceptible to predation [12]. Faster growth may also facilitate fledging or achieving mature size sooner [13], which can increase survival in the post-fledging period via faster acquisition of adult foraging capabilities [14], or more time spent foraging before the onset of winter [15]. However, in many species offspring do not grow at the maximum rate possible, indicating there may be some costs associated with fast growth [11]. Indeed, fast growth may come at the expense of resistance to oxidative stress or reductions in other immune functions [16,17]. Faster-growing individuals also may be disproportionately affected during times of resource limitation due to their greater absolute food requirements [12]. Additionally, faster-growing offspring may be more costly for parents to rear, and slower growth may be selected for when parents face difficult or unpredictable foraging conditions [18].

Dimorphism in growth rates may result in different selective pressures acting on males and females [19]. There is also growing evidence of plasticity in growth rates [20], and the degree of plasticity may vary between sexes in dimorphic species [21]. For example, in captive-reared Zebra Finches (*Taeniopygia guttata*), male nestlings grow at a faster rate than females at low feeding levels, but this pattern reversed at higher feeding levels [22]. Taken together, these factors indicate that a better understanding of the differences in growth rates between the sexes can aid our understanding of patterns of productivity in individuals and populations.

Here we examined, for the first time in Adélie Penguins (*Pygoscelis adeliae*), sex-based differences in chick mass and skeletal growth rates. We also evaluated the evidence for skewed sex ratio at hatching and investigated potential differences in diet between male and female chicks as explanations of observed differences in growth rates. The Adélie Penguin is a mildly dimorphic species with adult males averaging slightly larger than females in mass (mean ± SD = 5.0 ± 0.8 kg vs. 4.6 ± 0.7 kg), flipper length (210.9 ± 6.5 mm vs. 203.8 ± 6.8 mm) and bill length (35.6 ± 2.8 mm vs. 32.9 ± 2.4 mm; [23]). Adélie Penguin females lay 2 or occasionally 1 egg, and chicks hatch 1–3 days apart [24]. First-hatched chicks and those from 1-chick broods have been shown to grow faster than second-hatched chicks [24,25], but it remains unclear what effect chick sex may have on these relationships. Adélie Penguin chick mass growth follows a curvilinear pattern with peak mass reached between day 43–45 [25]. Male chicks are larger in mass but not skeletal size around the time of peak mass, and females but not males attain adult skeletal size by the time of fledging [26,27]. However, sex-based differences in growth rate have yet to be explicitly evaluated for this species. There are two primary components of Adélie Penguin diet in the southern Ross Sea, Crystal Krill (*Euphausia crystallorophias*) and Antarctic Silverfish (*Pleuragramma antarctica*), with Silverfish having a higher lipid content and thus offering higher quality diet to growing chicks [28]. Our analysis of diet differences in male and female chicks focused on differences in proportion of diet contributed by fish versus krill.

## Study Area and Methods

### Field work

Data were collected during the Austral summers of 2012–13 and 2013–14 (hereafter “2012” and “2013”, to reflect the year at the beginning of the summer) at Cape Crozier, Ross Island, Antarctica (77°31’S, 169°23’E). Cape Crozier is one of the largest colonies known for this species, with over 270,000 breeding pairs [29]. This study was part of a larger effort that used individually marked, known-age penguins to investigate reproductive ecology and demography (more info at www.penguinscience.com). The nests used in this study were chosen systematically from these known birds to represent a range of parental (i.e., age, breeding quality) and nest site (interior or edge of subcolonies) characteristics. Incubated nests were checked every 1–3 d to determine hatching day.

**Fig 1:**
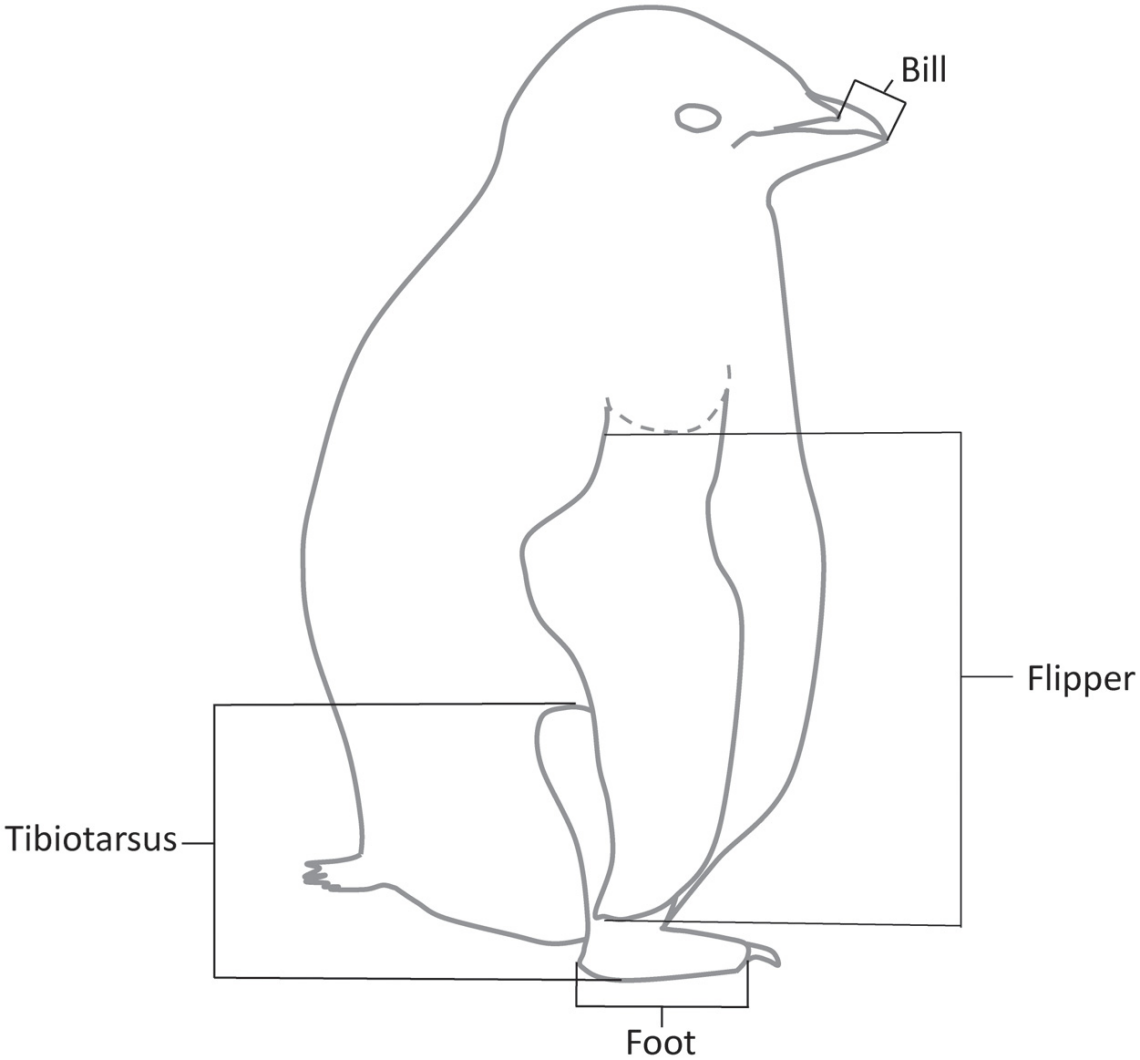
Measurement diagram. Schematic illustrating the location of morphological measurements collected from male and female Adélie Penguin chicks during Austral summers of 2012–13 and 2013–14 on Ross Island, Antarctica. Bill was measured to the nearest hundredth mm and remaining measurements to the nearest mm. Dashed line indicates humeral head on underside of the flipper.

Morphological measurements began at 10 d post-hatching for first-hatched chicks. Second-hatched chicks, if present, were also measured at this time, to reduce disturbance to the nest of returning just 1 or 2 d later. We collected five morphological measurements on chicks including: mass, measured to the nearest 25 g using a spring scale; bill length, measured (hundredth of a mm) with digital calipers from the most distal extent of skin on the side of the upper mandible to the tip of the bill; flipper length, measured along the underside of the flipper from the distal edge of the humeral head to the tip of the flipper; foot length, measured to the end of the longest (middle) toe excluding the toenail, with the tibiotarsus-tarsometatarsus joint held against the stop of a flush stop ruler; and tibiotarsus length, measured with the leg held in a natural position, with the femur-tibiotarsus joint held against the stop of a flush stop ruler, and measured to the sole of the foot (Fig 1). Flipper, foot, and tibiotarsus lengths were measured to the nearest mm. We collected 5 to 10 feathers (depending on feather size/plumage generation) from the cleft between the abdomen and leg on each chick for molecularly determining chick sex and for isotopic diet assessment. We collected these samples from two penguin chick plumages, one grown at 12–17 d and another grown at 25–35 d. We marked each chick with an individually numbered T-bar fish tag (Floy Tags Inc., USA) inserted subcutaneously at the back of the neck. Thereafter, we repeated morphological measurements on 5-d intervals for the remainder of the 50–55 d chick rearing period.

All penguin chick capture, handling, and data collection methods performed during this study were approved under appropriate Antarctic Conservation Act permits from the National Science Foundation Office of Polar Programs and Oregon State University’s Institutional Animal Care and Use Committee. Every effort was made to minimize the duration of human presence in the vicinity of any single study nest (generally <10 min), as well as handling time of individual penguins (<5 min).

### Analysis

Sex was determined using DNA extracted from the collected feathers [30]. We used a chi-squared test to evaluate evidence for unequal sex ratio at hatching between first- and second-hatched chicks in 2-chick broods, between 1- and 2-chick broods, and between years. We employed a 2-step process to model growth as a function of sex, while controlling for other factors. First, we fitted linear models to the measurements of each chick separately to obtain a daily growth rate for each chick. Adélie Penguin chick mass growth follows a sigmoidal shape, with a period of fast, linear growth between 10–40 d [25,31]. We fit linear models to the linear phase of growth only, which represented a different length of time for each morphological measurement (mass and flipper = 10–40 d post hatch; tibiotarsus and foot = 10–35 d; bill = 10–55 d). These periods of linear growth were determined by visual examination of fitted lines and comparison of R^2^ values for linear models fit to the growth data of each chick. For each morphological measurement, the time span was chosen that best encompassed the linear growth period for all chicks (i.e. R^2^ > 0.90 for most chicks, > 0.70 for all). We modeled the growth of each morphological parameter separately, and the slope coefficients from these models were taken as the daily growth rates for each morphological measurement for each chick.

We then modeled each morphological measurement separately by fitting linear, mixed-effects models with daily growth rate as the response variable and sex, hatching order (A or B), brood size (1 or 2) and year as fixed effects. Nests that initially had 2 chicks but subsequently lost one remained coded as 2-chick nests throughout the analysis. There was no clear pattern in timing of loss of a chick from 2-chick broods, and thus no clear criteria for reclassifying these nests part-way through the nesting season. These nests were retained in the analysis, despite the potential bias, in order to retain a sufficient sample sizes. However, it should be noted that most such losses occurred in second hatched chicks, and first hatched and single chicks had similar growth rates, so the bias associated with maintaining 2-chick brood coding was likely small. Nest ID was included as a random effect to account for lack of independence between siblings. We used random intercept only for the structure of our random effect, because this variable had too few levels (only 1 or 2 chicks per nest) to allow slope estimation. We examined the relative importance of the fixed effects by fitting candidate models with all possible main effects of the variables under consideration. Qualitative comparisons based on field observations (population level mean chick growth and reproductive success were lower during 2013–14 [32]) indicated possible differences in growth rates between the two years of the study, so we also considered models with the interactions between year and the remaining covariates. We used maximum-likelihood methods when determining best-supported fixed-effect model structure [33].

Upon determining the existence of sex-based differences in growth rates, we then evaluated the proportion of male versus female chick diet contributed by higher-lipid fish (versus krill) as a possible explanation for observed differences in chick growth rates. Adélie Penguin diet in the Southern Ross Sea is composed almost exclusively of some combination of Crystal Krill and Antarctic Silverfish, the latter of which contains a higher energy content than the former [34]. These prey items are separated by one trophic level (Silverfish feed on Crystal Krill), and thus the relative contribution of each prey type in the diet of penguin chicks can be determined via stable isotope analysis of penguin chick tissue [34]. We used stable isotope analysis of a portion of the feather samples to evaluate δ ^15^N isotope for each chick. Stable isotope values from feather material can provide information on diet during feather growth [35,36]. The ratio of ^15^N to ^14^N nitrogen isotopes in these feathers, relative to that in a standard sample (δ ^15^N; units as a proportion: ‰), was determined using a PDZ Europa ANCA-GSL elemental analyzer and a PDZ Europa 20–20 isotope ratio mass spectrometer (analysis conducted by the Stable Isotope Facility, UC Davis, USA). We focused on δ ^15^N because previous work has shown distinct differences in δ ^15^N values between fish and krill in this part of the southern ocean [37,38], and a relationship between δ ^15^N (but not δ ^13^C) and the proportion of fish in the diet has been detected at the colony scale on Ross Island [34]. We did not obtain sufficient feather samples from the plumage grown at 25–35 d to allow within-year comparison of diet between male and female chicks.

While it is theoretically possible (though not observed) that Adélie parents invest in male and female offspring of the same brood differently, Adelies do not vary the amount of food given to siblings [39] and it is unlikely that they could alter diet composition between male and female siblings. Therefor we restricted the analysis of diet to chicks from single-sex broods. To evaluate possible variability in δ ^15^N values between male and female chicks, we compared models with the main effects for sex and year alone, the additive sex and year effects and the interaction between year and sex, and also included the intercept-only model. In a preliminary analysis we included Nest ID as a random effect, because this was the random effect that was retained in the growth rate analysis. However, the variance estimated for Nest ID in the best-supported model was an order of magnitude smaller than the residual variance in the model, and thus we opted for a simpler and more easily interpretable analysis using fixed effects only.

In all analyses we compared Akaike information criterion values with a correction for small sample sizes (AIC_*c*_) and Akaike Weights (AIC_*c*_ wts) to evaluate models, and generally the model with the lowest AICc and highest AIC_*c*_ wt was selected as having the most support [40]. We evaluated the direction and strength of the effect of sex on growth by examining the estimated coefficients from the best-supported model and assessing whether 95% confidence limits overlapped zero. We calculated profile likelihood confidence intervals (CI) because the sampling distribution of variance estimates from mixed models may be asymmetric and sample sizes in this study were relatively small [33]. In all analyses the categorical covariates were coded as Sex: Male = 1 and Year: 2013 = 1. Analysis was conducted in R version 3.1.1 [41] and the package lme4 [42].

## Results

**Fig 2:**
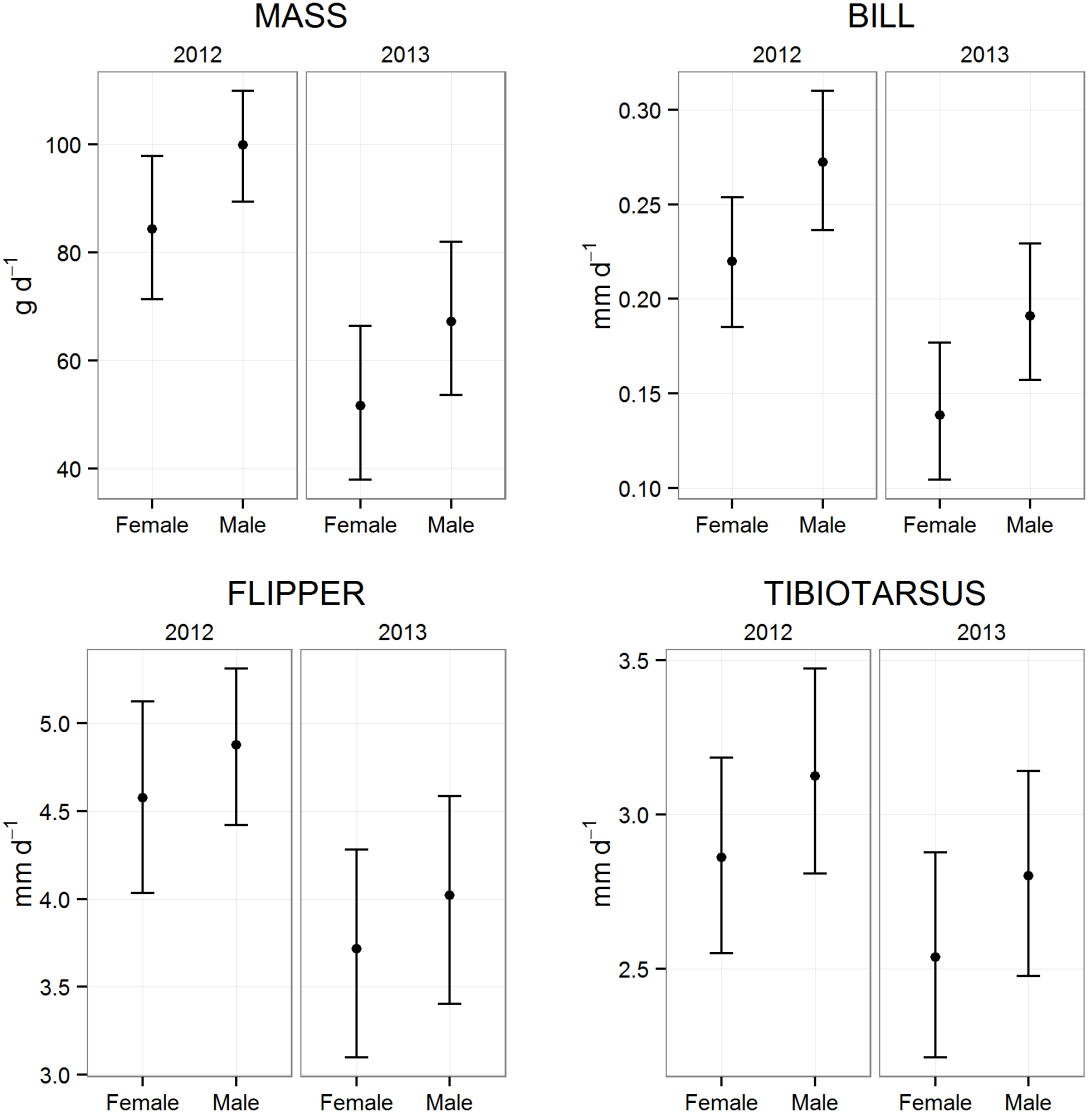
Estimated growth rates. Estimated average growth rates (with 95% CI) for mass, and length of bill, flipper and tibiotarsus for male and female Adélie Penguin chicks during 2012–13 (2012) and 2013–14 (2013) on Ross Island, Antarctica. Estimates calculated for mean values for other important variables in best model for each measurement (see text for details). Note different units and scales for y axes. Foot growth is not shown due to lack of support for an effect of sex.

We detected no differences in sex ratio by hatching order or brood size (Χ^2^ [2, N = 113 chicks] = 0.72; P = 0.20), or between seasons (Χ^2^ [1, N = 113 chicks] = 0.45; P = 0.50). The best-supported model for mass and flipper growth rates included Sex, Year, Hatching order, Brood size, and the interaction between Year and Hatching order (AIC_*c*_ wts. = 0.37 and 0.60, respectively). For mass growth, the 95% confidence interval on the Sex effect did not overlap zero, indicating that males gained mass at a faster rate than females in both years (Table 1). For flipper growth, there was only weak evidence for a positive effect of Sex on growth rate (*β* = 0.30; SE = 0.22; CI = -1.2 to 0.73; Table 2). The interaction between Year and Sex was not included in the best-supported model for any of the morphometric measurements, but it was included in 2 competitive (ΔAICc≤2) models for mass growth (ΔAICc = 0.91 and 1.1, respectively). However, in both of these models the 95% confidence interval for the coefficient of this Year*Sex interaction strongly overlapped 0 (*β* = -11.4; SE = 10.1; 95% CI = -31.7 to 8.6; and *β* = -12.0; SE = 10.2; 95% CI = -32.5 to 8.3, respectively). Thus while there was variation in overall chick growth rates between the 2 years of the study, there was little evidence that the difference between male and female growth rate varied between years (Fig 2).

**Table 1:** Growth model parameter estimates. Parameter estimates (with 95% CI) for the sex effect on growth from the best model (i.e., lowest AIC_c_) for each morphological measurement of Adélie Penguin chicks during 2012–13 and 2013–14 on Ross Island, Antarctica. Plus signs denote additive effects and asterisks denote interactions. Sex was not supported as an important factor associated with foot growth.

Morphological measurement	Best-supported model^a^	Estimate for sex effect	Lower 95% CI	Upper 95% CI
Mass (g d^-1^)	sex + brood size + hatch order*year	15.60	5.66	25.52
Flipper (mm d^-1^)	sex + year	0.30	-0.12	0.73
Bill (mm d^-1^)	sex + brood size + hatch order*year	0.05	0.02	0.09
Tibiotarsus (mm d^-1^)	sex + year	0.26	-0.08	0.60
Foot (mm d^-1^)	year	-	-	-

^a^ all models included nest ID as a random effect

**Table 2:** Estimated growth rates. Average daily growth rate estimates (with 95% CI) from best model (i.e., model with lowest AIC_c_) relating growth rates of morphological characteristics and mass to sex, year, brood size, chick hatching order; and sample sizes (n) for male and female Adélie Penguin chicks measured, and weighed during 2012–13 (2012) and 2013–14 (2013) on Ross Island, Antarctica. Sex was not supported as an important variable for foot growth so only means by year (best model results) are reported.

	2012		2013	
	Male	Female	Male	Female
n	23	21	37	32
Mass (g d^-1^)	99.89 (89.95–109.81)	84.29 (71.34–97.24)	67.24 (52.69–81.8)	51.64 (37.09–66.2)
Flipper (mm d^-1^)	4.88 (4.45–5.3)	4.58 (4.04–5.11)	4.02 (3.42–4.62)	3.72 (3.12–4.31)
Bill (mm d^-1^)	0.27 (0.24–0.31)	0.22 (0.19–0.25)	0.19 (0.15–0.23)	0.14 (0.10–0.18)
Tibiotarsus (mm d^-1^)	3.13 (2.78–3.47)	2.86 (2.54–3.19)	2.8 (2.45–3.15)	2.54 (2.19–2.89)
Foot (mm d^-1^)	1.06 (0.88–1.23)		1.27 (1.04–1.49)	

The best-supported model for bill and tibiotarsus growth included only Sex and Year (AIC_*c*_ weights = 0.53 and 0.40, respectively). For bill growth, there was strong evidence for faster growth in males (Table 1). However for tibiotarsus the 95% CI associated with the Sex coefficient slightly overlapped zero (Table 1), indicating only weak evidence for an effect. For foot growth, the best-supported model contained Year only (AIC_*c*_ wt. = 0.33).

While accounting for Year, Brood size and Hatching order, male Adélie Penguin chicks gained mass at an average rate of 15.6 g d^-1^ (95% CI: 5.6–25.5 g d^-1^) faster than females (Table 2, Fig 2). Across the duration of the linear growth period of mass (10 to 40 d) this dimorphism in growth rates led to a difference in estimated average size (at day 40) of 468 g. This estimated size difference is similar to the differences in observed mass of the few chicks we measured in the final days before fledging (13 males, 15 females), where males were on average 430 g heavier. Bill growth-rate (accounting for year) was also faster in males, and while the magnitude of the difference may seem relatively small (0.05 mm d^-1^, 95% CI: 0.02–0.09; Table 2, Fig 2), the percent difference in growth rates between males and females was actually greater for bill growth than mass growth. We found little evidence for an effect of sex on growth rates of flipper, tibiotarsus, or foot lengths (Tables 1 and 2, Fig 2).

**Fig 3:**
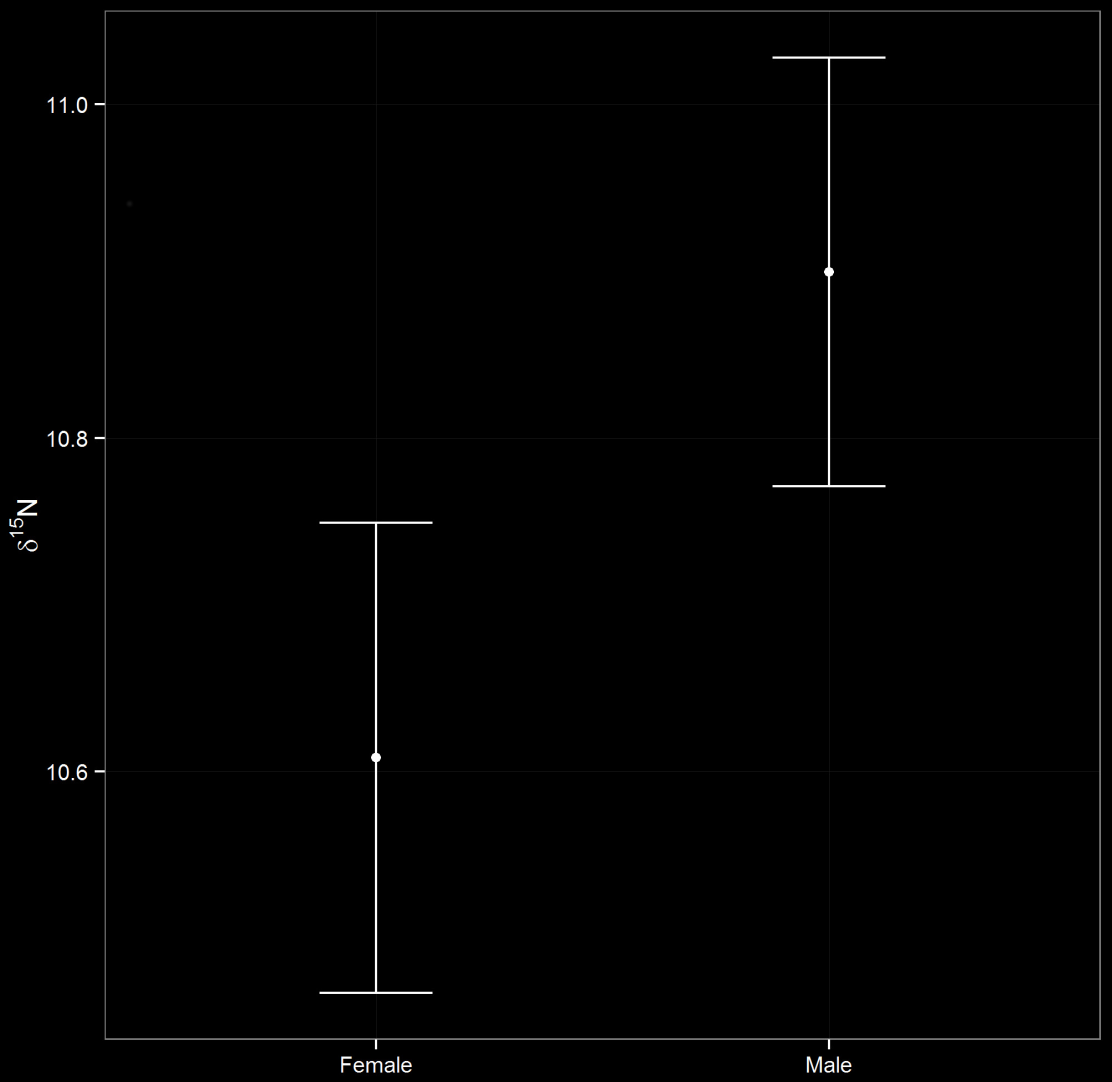
Sex-based differences in diet type. Estimated mean values for δ ^15^N (with 95% CI) for male and female Adélie Penguin chicks during 2012–13 and 2013–14 on Ross Island, Antarctica. δ ^15^N is a proxy for the proportion of fish in the diet.

In the investigation of the proportion of chick diet contributed by fish, the best-supported model contained just the main effect for sex. The coefficient for the sex effect in this model provided good evidence that male chicks were provisioned with more fish than female chicks, as indicated by the higher average δ ^15^N value in males (*β* = 0.29; 95% CI: 0.10 to 0.48; Fig 3). The second-ranked model contained the additive sex and year effects (ΔAICc = 1.4). However, since this model had one additional parameter but a ΔAICc value of approximately 2, we concluded that Year was an uninformative parameter [43], and that there was no support for year-based differences in the amount of fish fed to chicks (i.e., δ ^15^N).

## Discussion

We detected faster growth rates of mass and bill length in male Adélie Penguin chicks than in females. This difference in growth rates could have important implications for the reproductive ecology of this species, particularly at the very large colony where this study was conducted and where competition for food may be limiting colony growth and chick fledging sizes [28,29,44]. In the Antarctic Peninsula, an increase in fledging mass (at 50–55 d) of only 117 g, regardless of chick sex, increased the probability of recruitment in this species [45]. The difference in growth rates we observed was large enough to result in males being on average ~450 g heavier than females in the final 10 days before fledging. Thus, the difference we observed is likely to be biologically-significant, with the potential to cause differential survival between the sexes during both the pre-fledging and post-fledging periods. While chick sizes just before fledging observed in this study were smaller than those observed in other colonies on Ross Island and elsewhere in Antarctica, the relative degree of sexual dimorphism at this stage was similar to that observed elsewhere [26,28,44]

The rate of bill length growth between males and females also differed. Since bill growth was slow (0.22 mm d^-1^ for females), the observed 0.05 mm d^-1^ difference in growth rate was comparable to the difference in mass growth rates between the sexes, despite the seemingly small magnitude of this difference. Indeed, because bill growth continued for the duration of the 50–55 d chick-rearing period, unlike other skeletal measurements, by fledging age the bills of male chicks averaged 2.3 mm longer than those of females (as calculated from the best-supported model for bill growth; male = 24.4 mm, female = 21.9 mm). This difference is similar to the difference in average adult bill lengths at Cape Crozier [35.6 ± 2.8 mm vs. 32.9 ± 2.4 mm; 23], indicating that Adélie Penguin chicks nearly achieve adult dimorphism in bill size by fledging age. The growth of flipper, tibiotarsus, and foot length were all similar between males and females. However, there is dimorphism in flipper length in adults [23], and thus it seems likely that males continue growing flipper length longer than females.

Our analysis of diet composition revealed that the diet of male chicks had a greater proportion of fish than that of female chicks. The diet of adult and chick Adélie Penguins in the southern Ross Sea is composed almost entirely of Crystal Krill and Antarctic Silverfish [24]. Silverfish has a higher lipid content than krill and thus provide a more calorically-dense food for penguin chicks [34]. Thus, the difference in diet composition between male and female chicks is a possible mechanism for the sex-based differences in growth rates that we detected, and indeed a higher proportion of fish in the diet leads to more robust chicks regardless of sex [28]. This result provides some evidence of varying parental investment based on chick sex. Alternatively, parents may respond to the inherently higher growth rates of males by provisioning them with a greater proportion of fish [19]. Our study was unable to discriminate cause and effect in this relationship between diet type and growth rates, but nevertheless our results suggest that parents with only male chicks forage differently than parents with only female chicks, and that these differences are expressed in the differences in growth rates.

Regardless of the direction of cause and effect, the apparent variation in parental investment between male and female chicks may have implications for the condition of parents. Unlike other areas of the Southern Ocean where, owing to a generally narrow continental shelf, Silverfish are found near to shore and krill occupy offshore waters [46], in waters overlying the extensive continental shelf of the Ross Sea (where Crystal Krill is the dominant krill species, rather than Antarctic Krill) there is extensive horizontal overlap in distribution of these two main components of Adélie Penguin diet [34,47]. Thus, parents provisioning males with a greater proportion of fish are not required to travel greater distances to do so [26,48]. However, parents provisioning male chicks with more fish may nevertheless experience greater costs due to fish generally occurring at lower abundances and densities, and at greater depths [48] and having greater predator-avoidance capabilities [47,49–51]. Additionally, provisioning male chicks with a greater proportion of fish may influence the composition of diet retained by parents raising males versus females, thus further altering the difference in cost of raising chicks of the two sexes.

In Adélie Penguins, chicks hatch 1–2 d apart, and we observed that the first-hatched chick is often bigger and grows faster, possibly by outcompeting its sibling for food (also reported by [24]). Singleton chicks have also been reported to grow faster than those from 2-chick broods, likely due to greater overall access to food [25]. Given these patterns in growth, the results reported here could be due to the sex of first-hatched or singleton chicks being male biased. However, we detected no evidence of skewed sex ratios by hatching order or brood size, indicating that the differences in growth we report are not simply due to greater access to food for singleton chicks or a competitive advantage based solely on hatching-order-induced size differences.

The faster growth observed in male chicks may be a byproduct of adult SSD, rather than a feature providing a specific selective advantage during the growth phase. A rapid increase in both number and size of both fat and muscle cells is largely responsible for mass growth in Adélie Penguin chicks, with fat storage becoming more important and rapid in the second half of chick rearing [52]. The ability to both quickly deposit and later mobilize these fat reserves could provide selective advantage to individuals coping with such a short breeding season [52,53]. Considering our results in this context, it also may be that males need to have a greater capacity for increasing the mass of adipose tissue, which could benefit them later in life during several stages of reproductive effort when long-term fasting is required (more so in males than females [24]). Male Adélie Penguins arrive at the colony earlier in the season than females in order to secure territories, which allows for less pre-breeding foraging time and requires longer distance traveled via walking (vs. efficient swimming) over more extensive spring ice floes [54]. Males also take a larger proportion of the incubation duty, and generally lose a larger proportion of their mass during the breeding effort [55]. The development of fat cells in male penguin chicks may not necessarily provide any advantage during the growing period, but rather may be a developmental process expressed during growth that is important for breeding adult males later in life. Thus males may gain selective advantage in multiple ways by having a greater capacity for acquiring and storing energy reserves.

A little less than half of the nests in this study belonged to individual parents that were included in both years of the study. However, we did not account for this in our analysis due to limitations in sample size. Additionally, because over half of the nests belonged to parents that were only considered in one year, accounting for Parent ID in our analysis would have substantial overlap with the Nest ID covariate we included to account for possible similarities in growth rates between siblings. There is evidence that some individual parents consistently have higher reproductive success [56], but we felt it was more important to account for nestlings being siblings within a year than nestlings belonging to the same parents between years.

We have shown that offspring sex is an important factor in determining some components of growth rate in Adélie Penguin chicks (mass, bill length), and unimportant in others (flipper, tibiotarsus, foot). This is the first study to our knowledge to document sex-based differences in offspring growth rates in this species. Additionally, we found evidence that parents provided male offspring with higher quality diet than female offspring, indicating differential parental investment based on offspring sex. The identification of differences in provisioning and growth rates of male and female offspring provides a greater understanding of the ways in which ecological factors may impact the two sexes differently.
